# {μ-1,3-Bis[(3,5-dimethyl­pyrazol-1-yl)meth­yl]benzene-κ^2^ *N* ^2^:*N* ^2′^}di-μ-chlorido-bis­[chloridopalladium(II)] toluene solvate

**DOI:** 10.1107/S1600536809010629

**Published:** 2009-03-25

**Authors:** Bernard Omondi, Asheena Budhai, James Darkwa

**Affiliations:** a Department of Chemistry, University of Johannesburg, PO Box 524, Auckland Park, 2006 Johannesburg, South Africa

## Abstract

In the title complex, [Pd_2_Cl_4_(C_18_H_22_N_4_)]·C_7_H_8_, each of the two four-coordinated Pd^II^ atoms is in a slightly distorted square-planar geometry, defined by one N atom from the ligand, two bridging Cl atoms and one terminal Cl atom. Inter­molecular C—H⋯π inter­actions between the pyrazole ring H atom and the toluene ring stabilize the crystal structure.

## Related literature

For general background to poly(pyrazol-1-yl-meth­yl)benzene ligands and their palladium complexes, see: Hartshorn & Steel (1995 ▶, 1997 ▶, 1998 ▶); Motsoane *et al.* (2007 ▶); Yen *et al.* (2006 ▶). For related structures, see: Guzei *et al.* (2003 ▶).
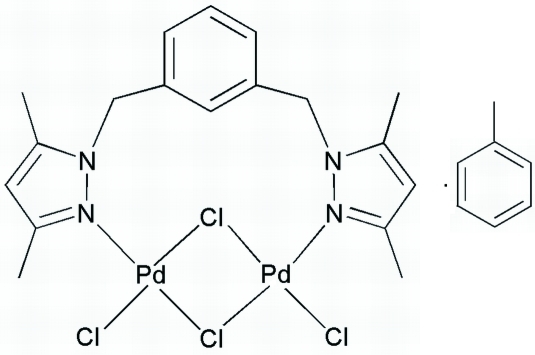


## Experimental

### 

#### Crystal data

[Pd_2_Cl_4_(C_18_H_22_N_4_)]·C_7_H_8_
*M* *_r_* = 741.13
Monoclinic, 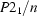

*a* = 10.4572 (10) Å
*b* = 25.376 (2) Å
*c* = 12.0782 (12) Å
β = 112.395 (4)°
*V* = 2963.4 (5) Å^3^
*Z* = 4
Mo *K*α radiation
μ = 1.60 mm^−1^
*T* = 298 K
0.50 × 0.12 × 0.06 mm

#### Data collection

Bruker SMART APEX CCD diffractometer
Absorption correction: multi-scan (*SADABS*; Bruker, 2001 ▶) *T*_min_ = 0.503, *T*_max_ = 0.910
22587 measured reflections
7158 independent reflections
4777 reflections with *I* > 2σ(*I*)
*R*_int_ = 0.045

#### Refinement

*R*[*F* ^2^ > 2σ(*F* ^2^)] = 0.042
*wR*(*F* ^2^) = 0.098
*S* = 1.02
7158 reflections
321 parameters
10 restraints
H-atom parameters constrained
Δρ_max_ = 0.53 e Å^−3^
Δρ_min_ = −0.58 e Å^−3^

### 

Data collection: *SMART* (Bruker, 2007 ▶); cell refinement: *SAINT* (Bruker, 2007 ▶); data reduction: *SAINT*; program(s) used to solve structure: *SHELXS97* (Sheldrick, 2008 ▶); program(s) used to refine structure: *SHELXL97* (Sheldrick, 2008 ▶); molecular graphics: *ORTEP-3* (Farrugia, 1997 ▶) and *DIAMOND* (Brandenburg, 1999 ▶); software used to prepare material for publication: *WinGX* (Farrugia, 1999 ▶).

## Supplementary Material

Crystal structure: contains datablocks global, I. DOI: 10.1107/S1600536809010629/hy2189sup1.cif

Structure factors: contains datablocks I. DOI: 10.1107/S1600536809010629/hy2189Isup2.hkl

Additional supplementary materials:  crystallographic information; 3D view; checkCIF report

## Figures and Tables

**Table 1:** Selected bond lengths (Å)

N2—Pd1	2.005 (3)
N4—Pd2	2.002 (3)
Cl1—Pd1	2.2647 (11)
Cl2—Pd2	2.2774 (12)
Cl3—Pd2	2.3421 (11)
Cl3—Pd1	2.3502 (10)
Cl4—Pd2	2.3092 (11)
Cl4—Pd1	2.3135 (12)

**Table 2:** Hydrogen-bond geometry (Å, °)

*D*—H⋯*A*	*D*—H	H⋯*A*	*D*⋯*A*	*D*—H⋯*A*
C16—H16⋯*Cg*1^i^	0.93	2.93	3.802 (6)	157

Symmetry code: (i) 
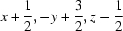
. *Cg*1 is the centroid of the C20–C25 ring.

**Fig. 1.**
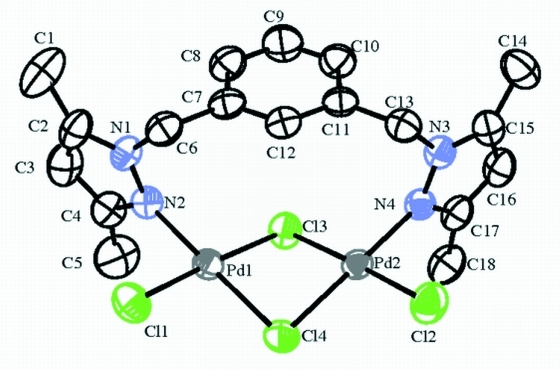
Molecular structure of the title compound. Displacement ellipsoids are drawn at the 50% probability level. H atoms and toluene molecule have been omitted for clarity.

**Fig. 2.**
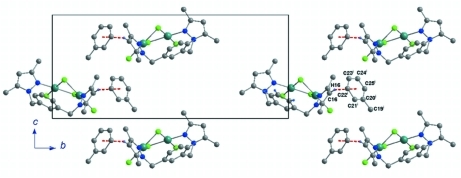
Packing diagram of the title compound, showing the intermolecular C—H···π interactions (dashed lines) linking the Pd complex and the toluene solvent molecule. [Symmetry code: (i) 1/2+x, 3/2-y, -1/2+z.]

## supplementary crystallographic information

### Comment

The title compound is of interest as part of a study of poly(pyrazol-1-yl-methyl)benzene palladium complexes as catalyst precursors for olefin oligomerization and polymerization. In a recent report (Motsoane *et al.*, 2007), coordination of Pd atom was shown to vary depending on the position of the pyrazol-1-yl-methyl group on the benzene linker. Poly(pyrazol-1-yl-methyl)benzene ligands can coordinate to Pd atoms through two independent pyrazolyl units (Motsoane *et al.*, 2007) or as a chelate ligand to a dinuclear unit with two bridging halides between the Pd atoms in a Pd_2_*X*_4_ (*X* = Cl) fashion (Yen *et al.*, 2006). This potential of poly(pyrazol-1-yl-methyl)benzene ligands exhibiting a variety of coordination modes was first reported in 1995 (Hartshorn & Steel, 1995). For the palladium complexes, two bonding modes have been reported. The first is a cage structure with six PdCl_2_ units and four 1,3,5-tris(pyrazol-1-yl-methyl)-2,4,6-triethylbenzene ligands, with coordination through the pyrazole N atoms (Hartshorn & Steel, 1997), and the second involves C—H activation, where coordination is through a pyrazole N atom as well as through the activated C atom (Hartshorn & Steel, 1998).

The title compound (Fig. 1) crystallizes from a mixture of chloroform and toluene and contains a dinuclear Pd complex molecule and a solvent toluene molecule in the asymmetric unit. The two Pd^II^ atoms are bridged by two Cl atoms. There are examples of similar structures in the literature, where the metal centers are bridged by halogen atoms (Cl or Br) (Guzei *et al.*, 2003; Motsoane *et al.*, 2007). Each of the Pd atoms has a distorted square-planar geometry (Table 1). The two square planes defined by the atoms around the Pd centers, N2, Cl1, Cl3, Cl4 for Pd1 and N4, Cl2, Cl3,Cl4 for Pd2, have a dihedral angle of 39.59 (1)° and atomic deviations from the planes of 0.018 and 0.011 Å, respectively. This dihedral angle results in a close contact between the two Pd centers [3.2116 (5) Å] and is probably due to steric bulk of the whole complex. The terminal as well as bridging Pd—Cl distances average 2.310 Å, which is close to the same distances of similar structues from the CSD (Guzei *et al.*, 2003; Motsoane *et al.*, 2007). The Pd—N bond distances [2.005 (3) and 2.002 (3) Å] are shorter than the corresponding distances from the CSD (2.1 (1) Å), as calculated by Guzei *et al.* (2003).

In the crystal structure, the dinuclear complex molecule is connected to the toluene molecule through a C—H···π interaction, with an H16···π distance of 2.93 Å (Fig. 2).

### Experimental

To a solution of PdCl_2_(NCMe)_2_ (0.44 g, 1.70 mmol) in CH_2_Cl_2_ (25 mL) was added 1,3-bis[(3,5-dimethylpyrazole-1-yl)methyl]benzene (0.50 g, 1.70 mmol). The resultant solution was stired overnight, and after removal of solvent, a dark orange solid was obtained. Recrystallization was done in a mixture of CHCl_3_ and toluene, giving needle-shaped crystals.

### Refinement

H atoms were positioned geometrically and refined as riding atoms, with C—H = 0.93 (aromatic), 0.97 (CH_2_) and 0.96 (CH_3_) Å and with *U*_iso_(H) = 1.2(or 1.5 for methyl)*U*_eq_(C).

### Crystal data

**Table:**

[Pd_2_Cl_4_(C_18_H_22_N_4_)]·C_7_H_8_	*F*(000) = 1472
*M**_r_* = 741.13	*D*_x_ = 1.661 Mg m^−^^3^
Monoclinic, *P*2_1_/*n*	Mo *K*α radiation, λ = 0.71073 Å
Hall symbol: -P 2yn	Cell parameters from 22587 reflections
*a* = 10.4572 (10) Å	θ = 2.0–28.0°
*b* = 25.376 (2) Å	µ = 1.60 mm^−^^1^
*c* = 12.0782 (12) Å	*T* = 298 K
β = 112.395 (4)°	Needle, brown
*V* = 2963.4 (5) Å^3^	0.50 × 0.12 × 0.06 mm
*Z* = 4	

### Data collection

**Table:**

Bruker SMART APEX CCD diffractometer	7158 independent reflections
Radiation source: fine-focus sealed tube	4777 reflections with *I* > 2σ(*I*)
graphite	*R*_int_ = 0.045
φ and ω scans	θ_max_ = 28.0°, θ_min_ = 2.0°
Absorption correction: multi-scan (*SADABS*; Bruker, 2001)	*h* = −13→13
*T*_min_ = 0.503, *T*_max_ = 0.910	*k* = −24→33
22587 measured reflections	*l* = −10→15

### Refinement

**Table:**

Refinement on *F*^2^	10 restraints
Least-squares matrix: full	H-atom parameters constrained
*R*[*F*^2^ > 2σ(*F*^2^)] = 0.042	*w* = 1/[σ^2^(*F*_o_^2^) + (0.0445*P*)^2^ + 1.0406*P*] where *P* = (*F*_o_^2^ + 2*F*_c_^2^)/3
*wR*(*F*^2^) = 0.098	(Δ/σ)_max_ = 0.012
*S* = 1.02	Δρ_max_ = 0.53 e Å^−^^3^
7158 reflections	Δρ_min_ = −0.57 e Å^−^^3^
321 parameters	

### Fractional atomic coordinates and isotropic or equivalent isotropic displacement parameters (Å^2^)

**Table:**

	*x*	*y*	*z*	*U*_iso_*/*U*_eq_	
C1	0.1871 (7)	0.8038 (2)	0.2496 (6)	0.099 (2)	
H1A	0.1059	0.8010	0.1775	0.149*	
H1B	0.1931	0.7738	0.2996	0.149*	
H1C	0.2676	0.8052	0.2297	0.149*	
C2	0.1790 (5)	0.85270 (18)	0.3147 (5)	0.0595 (13)	
C3	0.1918 (5)	0.8599 (2)	0.4306 (5)	0.0695 (14)	
H3	0.2075	0.8337	0.4882	0.083*	
C4	0.1771 (4)	0.9140 (2)	0.4470 (4)	0.0571 (11)	
C5	0.1804 (7)	0.9431 (2)	0.5530 (5)	0.0889 (18)	
H5A	0.1983	0.9797	0.5444	0.133*	
H5B	0.2522	0.9291	0.6231	0.133*	
H5C	0.0928	0.9396	0.5609	0.133*	
C6	0.1524 (4)	0.91608 (18)	0.1445 (4)	0.0514 (11)	
H6A	0.0709	0.9376	0.1051	0.062*	
H6B	0.1445	0.8848	0.0963	0.062*	
C7	0.2807 (4)	0.94690 (18)	0.1525 (4)	0.0471 (11)	
C8	0.4127 (5)	0.9274 (2)	0.2212 (5)	0.0604 (13)	
H8	0.4225	0.8955	0.2615	0.073*	
C9	0.5274 (5)	0.9558 (2)	0.2283 (5)	0.0695 (15)	
H9	0.6151	0.9428	0.2727	0.083*	
C10	0.5141 (4)	1.0033 (2)	0.1707 (5)	0.0576 (13)	
H10	0.5928	1.0220	0.1764	0.069*	
C11	0.3838 (4)	1.02362 (17)	0.1036 (4)	0.0450 (10)	
C12	0.2680 (4)	0.99412 (17)	0.0948 (4)	0.0433 (10)	
H12	0.1803	1.0068	0.0488	0.052*	
C13	0.3681 (4)	1.07703 (17)	0.0446 (4)	0.0494 (11)	
H13A	0.4033	1.0755	−0.0189	0.059*	
H13B	0.2709	1.0862	0.0090	0.059*	
C14	0.6392 (5)	1.1343 (2)	0.0648 (5)	0.0722 (15)	
H14A	0.6685	1.0983	0.0691	0.108*	
H14B	0.7187	1.1570	0.0893	0.108*	
H14C	0.5796	1.1426	−0.0160	0.108*	
C15	0.5632 (4)	1.14228 (18)	0.1452 (4)	0.0493 (11)	
C16	0.5951 (4)	1.17376 (19)	0.2460 (4)	0.0579 (13)	
H16	0.6714	1.1958	0.2780	0.070*	
C17	0.4926 (4)	1.16631 (17)	0.2904 (4)	0.0501 (11)	
C18	0.4749 (5)	1.1888 (2)	0.3991 (5)	0.0672 (14)	
H18A	0.3803	1.1991	0.3784	0.101*	
H18B	0.5339	1.2189	0.4271	0.101*	
H18C	0.4993	1.1626	0.4611	0.101*	
N1	0.1581 (3)	0.90068 (14)	0.2629 (3)	0.0473 (9)	
N2	0.1594 (3)	0.93824 (14)	0.3438 (3)	0.0446 (8)	
N3	0.4434 (3)	1.11796 (13)	0.1316 (3)	0.0453 (8)	
N4	0.3997 (3)	1.13181 (13)	0.2198 (3)	0.0435 (8)	
Cl1	−0.12855 (10)	0.97616 (5)	0.21818 (11)	0.0614 (3)	
Cl2	0.11537 (12)	1.17589 (5)	0.07665 (12)	0.0656 (3)	
Cl3	0.30569 (10)	1.05135 (4)	0.37297 (10)	0.0524 (3)	
Cl4	0.00312 (10)	1.09512 (5)	0.23267 (12)	0.0601 (3)	
Pd1	0.08672 (3)	1.011252 (13)	0.29434 (3)	0.04228 (10)	
Pd2	0.21405 (3)	1.114894 (13)	0.22275 (3)	0.04230 (10)	
C19	0.2046 (12)	0.1522 (5)	0.5868 (12)	0.230 (7)	
H19A	0.1707	0.1732	0.5154	0.345*	
H19B	0.1281	0.1360	0.5992	0.345*	
H19C	0.2650	0.1253	0.5785	0.345*	
C20	0.2793 (10)	0.1850 (4)	0.6875 (11)	0.136 (3)	
C21	0.3754 (11)	0.2193 (4)	0.6843 (11)	0.147 (4)	
H21	0.3940	0.2233	0.6154	0.177*	
C22	0.4488 (12)	0.2496 (5)	0.7898 (14)	0.165 (5)	
H22	0.5158	0.2736	0.7892	0.198*	
C23	0.4229 (9)	0.2439 (4)	0.8855 (11)	0.149 (4)	
H23	0.4704	0.2636	0.9538	0.179*	
C24	0.3204 (9)	0.2070 (4)	0.8840 (9)	0.126 (3)	
H24	0.3032	0.2030	0.9536	0.151*	
C25	0.2452 (9)	0.1769 (3)	0.7868 (10)	0.129 (3)	
H25	0.1775	0.1533	0.7872	0.155*	

### Atomic displacement parameters (Å^2^)

**Table:**

	*U*^11^	*U*^22^	*U*^33^	*U*^12^	*U*^13^	*U*^23^
C1	0.134 (6)	0.049 (4)	0.098 (5)	0.006 (3)	0.026 (4)	−0.002 (3)
C2	0.060 (3)	0.039 (3)	0.067 (4)	0.002 (2)	0.011 (2)	0.008 (3)
C3	0.068 (3)	0.058 (2)	0.067 (4)	0.004 (2)	0.009 (3)	0.022 (3)
C4	0.054 (2)	0.063 (2)	0.046 (2)	0.003 (2)	0.010 (2)	0.012 (2)
C5	0.120 (5)	0.093 (5)	0.054 (3)	0.018 (4)	0.033 (3)	0.010 (3)
C6	0.055 (2)	0.049 (3)	0.045 (3)	0.0004 (19)	0.013 (2)	−0.007 (2)
C7	0.046 (2)	0.049 (3)	0.041 (2)	0.0039 (18)	0.0108 (18)	−0.011 (2)
C8	0.060 (3)	0.052 (3)	0.065 (3)	0.009 (2)	0.019 (2)	0.004 (3)
C9	0.047 (2)	0.074 (4)	0.081 (4)	0.016 (2)	0.016 (2)	0.013 (3)
C10	0.044 (2)	0.060 (3)	0.065 (3)	0.003 (2)	0.016 (2)	−0.002 (3)
C11	0.045 (2)	0.049 (3)	0.039 (2)	0.0046 (18)	0.0148 (17)	−0.006 (2)
C12	0.0423 (19)	0.048 (3)	0.033 (2)	0.0029 (17)	0.0066 (16)	−0.006 (2)
C13	0.047 (2)	0.055 (3)	0.046 (3)	−0.0024 (19)	0.0171 (19)	−0.006 (2)
C14	0.055 (3)	0.091 (4)	0.076 (4)	0.001 (3)	0.031 (3)	0.012 (3)
C15	0.041 (2)	0.054 (3)	0.048 (3)	0.0002 (19)	0.0114 (18)	0.008 (2)
C16	0.045 (2)	0.051 (3)	0.065 (3)	−0.0112 (19)	0.006 (2)	0.000 (3)
C17	0.047 (2)	0.041 (3)	0.051 (3)	−0.0023 (18)	0.0063 (19)	−0.001 (2)
C18	0.077 (3)	0.052 (3)	0.059 (3)	−0.009 (2)	0.011 (3)	−0.015 (3)
N1	0.0525 (19)	0.038 (2)	0.047 (2)	−0.0025 (15)	0.0139 (16)	−0.0023 (18)
N2	0.0427 (17)	0.047 (2)	0.040 (2)	−0.0013 (15)	0.0111 (15)	0.0000 (18)
N3	0.0402 (16)	0.043 (2)	0.047 (2)	−0.0019 (14)	0.0114 (15)	0.0005 (17)
N4	0.0421 (16)	0.041 (2)	0.046 (2)	−0.0030 (14)	0.0150 (15)	−0.0042 (17)
Cl1	0.0409 (5)	0.0746 (9)	0.0621 (8)	−0.0089 (5)	0.0123 (5)	−0.0036 (6)
Cl2	0.0581 (6)	0.0569 (8)	0.0727 (9)	0.0068 (5)	0.0148 (6)	0.0169 (7)
Cl3	0.0417 (5)	0.0538 (7)	0.0524 (7)	−0.0020 (4)	0.0074 (4)	0.0048 (5)
Cl4	0.0436 (5)	0.0561 (7)	0.0845 (9)	0.0107 (5)	0.0286 (5)	0.0119 (7)
Pd1	0.03703 (15)	0.0461 (2)	0.04121 (19)	0.00065 (13)	0.01216 (13)	0.00031 (16)
Pd2	0.03750 (15)	0.03964 (19)	0.0467 (2)	0.00144 (12)	0.01255 (13)	−0.00171 (15)
C19	0.199 (12)	0.186 (12)	0.217 (13)	0.093 (10)	−0.019 (10)	−0.040 (11)
C20	0.126 (7)	0.115 (7)	0.167 (9)	0.043 (5)	0.056 (6)	0.040 (7)
C21	0.153 (9)	0.128 (9)	0.208 (11)	0.062 (5)	0.122 (9)	0.070 (7)
C22	0.174 (11)	0.131 (9)	0.249 (14)	0.052 (7)	0.145 (11)	0.050 (8)
C23	0.118 (6)	0.126 (7)	0.220 (11)	0.052 (4)	0.082 (7)	0.070 (8)
C24	0.115 (6)	0.116 (7)	0.167 (8)	0.061 (4)	0.076 (6)	0.075 (6)
C25	0.116 (6)	0.091 (6)	0.187 (9)	0.054 (5)	0.065 (6)	0.068 (6)

### Geometric parameters (Å, °)

**Table:**

C1—C2	1.488 (8)	C15—N3	1.349 (5)
C1—H1A	0.9600	C15—C16	1.387 (6)
C1—H1B	0.9600	C16—C17	1.381 (7)
C1—H1C	0.9600	C16—H16	0.9300
C2—N1	1.348 (6)	C17—N4	1.344 (5)
C2—C3	1.367 (7)	C17—C18	1.506 (7)
C3—C4	1.403 (7)	C18—H18A	0.9600
C3—H3	0.9300	C18—H18B	0.9600
C4—N2	1.339 (6)	C18—H18C	0.9600
C4—C5	1.468 (7)	N1—N2	1.361 (5)
C5—H5A	0.9600	N2—Pd1	2.005 (3)
C5—H5B	0.9600	N3—N4	1.356 (5)
C5—H5C	0.9600	N4—Pd2	2.002 (3)
C6—N1	1.462 (6)	Cl1—Pd1	2.2647 (11)
C6—C7	1.524 (6)	Cl2—Pd2	2.2774 (12)
C6—H6A	0.9700	Cl3—Pd2	2.3421 (11)
C6—H6B	0.9700	Cl3—Pd1	2.3502 (10)
C7—C12	1.367 (6)	Cl4—Pd2	2.3092 (11)
C7—C8	1.402 (6)	Cl4—Pd1	2.3135 (12)
C8—C9	1.375 (7)	Pd1—Pd2	3.2117 (5)
C8—H8	0.9300	C19—C20	1.435 (11)
C9—C10	1.371 (7)	C19—H19A	0.9600
C9—H9	0.9300	C19—H19B	0.9600
C10—C11	1.393 (6)	C19—H19C	0.9600
C10—H10	0.9300	C20—C21	1.341 (13)
C11—C12	1.393 (6)	C20—C25	1.391 (13)
C11—C13	1.511 (6)	C21—C22	1.435 (15)
C12—H12	0.9300	C21—H21	0.9300
C13—N3	1.474 (5)	C22—C23	1.292 (13)
C13—H13A	0.9700	C22—H22	0.9300
C13—H13B	0.9700	C23—C24	1.418 (12)
C14—C15	1.484 (7)	C23—H23	0.9300
C14—H14A	0.9600	C24—C25	1.370 (12)
C14—H14B	0.9600	C24—H24	0.9300
C14—H14C	0.9600	C25—H25	0.9300
			
C2—C1—H1A	109.5	C16—C17—C18	131.1 (4)
C2—C1—H1B	109.5	C17—C18—H18A	109.5
H1A—C1—H1B	109.5	C17—C18—H18B	109.5
C2—C1—H1C	109.5	H18A—C18—H18B	109.5
H1A—C1—H1C	109.5	C17—C18—H18C	109.5
H1B—C1—H1C	109.5	H18A—C18—H18C	109.5
N1—C2—C3	106.9 (4)	H18B—C18—H18C	109.5
N1—C2—C1	122.6 (5)	C2—N1—N2	110.1 (4)
C3—C2—C1	130.5 (5)	C2—N1—C6	129.4 (4)
C2—C3—C4	107.6 (5)	N2—N1—C6	120.0 (3)
C2—C3—H3	126.2	C4—N2—N1	107.8 (4)
C4—C3—H3	126.2	C4—N2—Pd1	127.2 (3)
N2—C4—C3	107.6 (5)	N1—N2—Pd1	122.3 (3)
N2—C4—C5	122.0 (5)	C15—N3—N4	111.1 (3)
C3—C4—C5	130.5 (5)	C15—N3—C13	128.9 (4)
C4—C5—H5A	109.5	N4—N3—C13	119.6 (3)
C4—C5—H5B	109.5	C17—N4—N3	106.8 (3)
H5A—C5—H5B	109.5	C17—N4—Pd2	126.8 (3)
C4—C5—H5C	109.5	N3—N4—Pd2	125.3 (2)
H5A—C5—H5C	109.5	Pd2—Cl3—Pd1	86.39 (3)
H5B—C5—H5C	109.5	Pd2—Cl4—Pd1	88.01 (4)
N1—C6—C7	111.6 (3)	N2—Pd1—Cl1	87.88 (10)
N1—C6—H6A	109.3	N2—Pd1—Cl4	178.57 (11)
C7—C6—H6A	109.3	Cl1—Pd1—Cl4	92.05 (4)
N1—C6—H6B	109.3	N2—Pd1—Cl3	94.55 (9)
C7—C6—H6B	109.3	Cl1—Pd1—Cl3	177.47 (5)
H6A—C6—H6B	108.0	Cl4—Pd1—Cl3	85.54 (4)
C12—C7—C8	119.7 (4)	N2—Pd1—Pd2	133.45 (9)
C12—C7—C6	120.3 (4)	Cl1—Pd1—Pd2	131.49 (4)
C8—C7—C6	120.0 (4)	Cl4—Pd1—Pd2	45.94 (3)
C9—C8—C7	119.3 (5)	Cl3—Pd1—Pd2	46.70 (3)
C9—C8—H8	120.4	N4—Pd2—Cl2	89.80 (10)
C7—C8—H8	120.4	N4—Pd2—Cl4	178.19 (11)
C10—C9—C8	120.9 (4)	Cl2—Pd2—Cl4	91.61 (4)
C10—C9—H9	119.6	N4—Pd2—Cl3	92.76 (10)
C8—C9—H9	119.6	Cl2—Pd2—Cl3	177.44 (4)
C9—C10—C11	120.5 (4)	Cl4—Pd2—Cl3	85.82 (4)
C9—C10—H10	119.7	N4—Pd2—Pd1	133.22 (10)
C11—C10—H10	119.7	Cl2—Pd2—Pd1	130.78 (3)
C12—C11—C10	118.4 (4)	Cl4—Pd2—Pd1	46.05 (3)
C12—C11—C13	120.8 (4)	Cl3—Pd2—Pd1	46.91 (3)
C10—C11—C13	120.8 (4)	C20—C19—H19A	109.5
C7—C12—C11	121.3 (4)	C20—C19—H19B	109.5
C7—C12—H12	119.4	H19A—C19—H19B	109.5
C11—C12—H12	119.4	C20—C19—H19C	109.5
N3—C13—C11	111.3 (3)	H19A—C19—H19C	109.5
N3—C13—H13A	109.4	H19B—C19—H19C	109.5
C11—C13—H13A	109.4	C21—C20—C19	121.7 (13)
N3—C13—H13B	109.4	C21—C20—C25	124.1 (12)
C11—C13—H13B	109.4	C19—C20—C25	114.2 (12)
H13A—C13—H13B	108.0	C20—C21—C22	118.4 (12)
C15—C14—H14A	109.5	C20—C21—H21	120.8
C15—C14—H14B	109.5	C22—C21—H21	120.8
H14A—C14—H14B	109.5	C23—C22—C21	120.7 (13)
C15—C14—H14C	109.5	C23—C22—H22	119.6
H14A—C14—H14C	109.5	C21—C22—H22	119.6
H14B—C14—H14C	109.5	C22—C23—C24	118.5 (13)
N3—C15—C16	105.8 (4)	C22—C23—H23	120.7
N3—C15—C14	124.2 (4)	C24—C23—H23	120.7
C16—C15—C14	130.1 (4)	C25—C24—C23	124.3 (11)
C17—C16—C15	107.4 (4)	C25—C24—H24	117.9
C17—C16—H16	126.3	C23—C24—H24	117.9
C15—C16—H16	126.3	C24—C25—C20	113.9 (10)
N4—C17—C16	108.9 (4)	C24—C25—H25	123.0
N4—C17—C18	120.0 (4)	C20—C25—H25	123.0
			
N1—C2—C3—C4	0.3 (5)	C15—N3—N4—C17	0.9 (5)
C1—C2—C3—C4	179.3 (5)	C13—N3—N4—C17	175.2 (3)
C2—C3—C4—N2	−1.5 (5)	C15—N3—N4—Pd2	169.8 (3)
C2—C3—C4—C5	179.3 (5)	C13—N3—N4—Pd2	−15.8 (5)
N1—C6—C7—C12	−129.2 (4)	C4—N2—Pd1—Cl1	−88.8 (3)
N1—C6—C7—C8	49.6 (6)	N1—N2—Pd1—Cl1	70.3 (3)
C12—C7—C8—C9	−0.7 (7)	C4—N2—Pd1—Cl3	90.5 (3)
C6—C7—C8—C9	−179.5 (5)	N1—N2—Pd1—Cl3	−110.3 (3)
C7—C8—C9—C10	1.0 (8)	C4—N2—Pd1—Pd2	119.8 (3)
C8—C9—C10—C11	0.0 (8)	N1—N2—Pd1—Pd2	−81.1 (3)
C9—C10—C11—C12	−1.2 (7)	Pd2—Cl4—Pd1—Cl1	−152.62 (5)
C9—C10—C11—C13	177.1 (4)	Pd2—Cl4—Pd1—Cl3	28.14 (4)
C8—C7—C12—C11	−0.6 (6)	Pd2—Cl3—Pd1—N2	150.81 (11)
C6—C7—C12—C11	178.2 (4)	Pd2—Cl3—Pd1—Cl4	−27.75 (4)
C10—C11—C12—C7	1.5 (6)	C17—N4—Pd2—Cl2	94.0 (4)
C13—C11—C12—C7	−176.8 (4)	N3—N4—Pd2—Cl2	−72.8 (3)
C12—C11—C13—N3	125.1 (4)	C17—N4—Pd2—Cl3	−86.0 (3)
C10—C11—C13—N3	−53.2 (5)	N3—N4—Pd2—Cl3	107.2 (3)
N3—C15—C16—C17	0.9 (5)	C17—N4—Pd2—Pd1	−112.8 (3)
C14—C15—C16—C17	−178.7 (5)	N3—N4—Pd2—Pd1	80.5 (3)
C15—C16—C17—N4	−0.4 (5)	Pd1—Cl4—Pd2—Cl2	151.71 (5)
C15—C16—C17—C18	178.1 (5)	Pd1—Cl4—Pd2—Cl3	−28.24 (4)
C3—C2—N1—N2	0.9 (5)	Pd1—Cl3—Pd2—N4	−153.33 (10)
C1—C2—N1—N2	−178.2 (5)	Pd1—Cl3—Pd2—Cl4	27.80 (4)
C3—C2—N1—C6	172.8 (4)	N2—Pd1—Pd2—N4	−4.1 (2)
C1—C2—N1—C6	−6.3 (7)	Cl1—Pd1—Pd2—N4	−144.43 (15)
C7—C6—N1—C2	−107.5 (5)	Cl4—Pd1—Pd2—N4	177.72 (15)
C7—C6—N1—N2	63.6 (5)	Cl3—Pd1—Pd2—N4	37.97 (14)
C3—C4—N2—N1	2.0 (5)	N2—Pd1—Pd2—Cl2	139.48 (15)
C5—C4—N2—N1	−178.7 (4)	Cl1—Pd1—Pd2—Cl2	−0.88 (7)
C3—C4—N2—Pd1	163.6 (3)	Cl4—Pd1—Pd2—Cl2	−38.73 (7)
C5—C4—N2—Pd1	−17.1 (6)	Cl3—Pd1—Pd2—Cl2	−178.48 (7)
C2—N1—N2—C4	−1.9 (4)	N2—Pd1—Pd2—Cl4	178.21 (15)
C6—N1—N2—C4	−174.6 (3)	Cl1—Pd1—Pd2—Cl4	37.85 (7)
C2—N1—N2—Pd1	−164.5 (3)	Cl3—Pd1—Pd2—Cl4	−139.75 (6)
C6—N1—N2—Pd1	22.7 (4)	N2—Pd1—Pd2—Cl3	−42.04 (14)
C16—C15—N3—N4	−1.1 (5)	Cl1—Pd1—Pd2—Cl3	177.61 (6)
C14—C15—N3—N4	178.5 (4)	Cl4—Pd1—Pd2—Cl3	139.75 (7)
C16—C15—N3—C13	−174.8 (4)	C19—C20—C21—C22	−178.1 (9)
C14—C15—N3—C13	4.9 (7)	C25—C20—C21—C22	0.3 (14)
C11—C13—N3—C15	105.7 (5)	C20—C21—C22—C23	0.0 (16)
C11—C13—N3—N4	−67.5 (4)	C21—C22—C23—C24	0.1 (15)
C16—C17—N4—N3	−0.3 (5)	C22—C23—C24—C25	−0.6 (13)
C18—C17—N4—N3	−178.9 (4)	C23—C24—C25—C20	0.9 (12)
C16—C17—N4—Pd2	−169.0 (3)	C21—C20—C25—C24	−0.8 (12)
C18—C17—N4—Pd2	12.3 (6)	C19—C20—C25—C24	177.8 (8)

### Hydrogen-bond geometry (Å, °)

**Table:**

*D*—H···*A*	*D*—H	H···*A*	*D*···*A*	*D*—H···*A*
C16—H16···Cg1^i^	0.93	2.93	3.802 (6)	157

Symmetry codes: (i) *x*+1/2, −*y*+3/2, *z*−1/2.
